# Dynamic changes of Kisspeptin during controlled ovarian hyperstimulation (COH) in polycystic ovary syndrome patients and its correlation with COH outcomes

**DOI:** 10.3389/fendo.2026.1835699

**Published:** 2026-05-20

**Authors:** Lu Han, Yiran Wang, Yueying Li, Xiaoyan Li, Jing Zhang, Yan Zhang, Mingfei Zhao, Haixia Chen, Huiying Zhang, Yingmei Wang, Fengxia Xue, Wenyan Tian

**Affiliations:** 1 Department of Gynecology and Obstetrics, Tianjin Medical University General Hospital, Tianjin, China; 2 Tianjin Key Laboratory of Female Reproductive Health and Eugenic, Tianjin Medical University General Hospital, Tianjin, China

**Keywords:** controlled ovarian hyperstimulation, Kisspeptin, live birth, polycystic ovary syndrome, pregnancy

## Abstract

**Background:**

Polycystic ovary syndrome (PCOS) is a common cause of anovulatory infertility in women, and patients often exhibit ovarian hyperresponse during controlled ovarian hyperstimulation (COH) in assisted reproductive technology. Kisspeptin is a key upstream regulator of the hypothalamic-pituitary-ovarian axis (HPOA). This study aims to explore the dynamic changes in serum and follicular fluid (FF) Kisspeptin levels during COH in PCOS patients and analyze their correlation with COH outcomes and pregnancy outcomes, in order to evaluate the potential value of Kisspeptin as a predictive biomarker.

**Methods:**

This prospective cohort study included 100 patients undergoing IVF-ET treatment (50 in the PCOS group and 50 in the control group). Serum samples were collected at four time points during the COH cycle (the day of starting Gn treatment, the fifth day of Gn stimulation, the day of hCG injection, the day of oocyte pickup [dOPU]), and FF samples were obtained on dOPU. Baseline characteristics, Kisspeptin levels, COH and pregnancy outcomes were compared between groups. Correlation and ROC curve analyses were performed.

**Results:**

Serum and FF Kisspeptin levels were significantly higher in the PCOS group at all time points (P<0.001). Both groups showed significantly elevated Kisspeptin levels on dOPU compared with dGn (P<0.001). Correlation analysis revealed that serum and FF Kisspeptin levels were positively correlated with AFC, AMH, E2 on dhCG, number of retrieved oocytes, and number of available embryos, but negatively correlated with the top-quality embryo (TQE) rate (P<0.01). Although the PCOS group had higher numbers of retrieved oocytes and available embryos, their TQE rate, cumulative pregnancy rate (40.00% vs. 60.00%, P = 0.046), and live birth rate (8.54% vs. 35.06%, P = 0.014) were significantly lower than those of the control group. ROC curve analysis indicated that serum Kisspeptin levels on dOPU had a certain predictive value for pregnancy outcomes (AUC = 0.655, 95% CI: 0.547–0.763).

**Conclusion:**

Kisspeptin may be involved in the pathological processes of ovarian hyperresponse and asynchronous follicular development in PCOS and possesses a certain predictive value for pregnancy outcomes. It provides an important basis for understanding the mechanisms of reproductive dysfunction in PCOS and for developing future multifactorial predictive models.

## 1 Introduction

Polycystic ovary syndrome (PCOS) is a common disorder characterized by reproductive, endocrine, and metabolic abnormalities, primarily affecting women of reproductive age (1). Its core features include hyperandrogenemia, ovulatory dysfunction (such as oligomenorrhea or amenorrhea), and polycystic ovarian morphology, making it a leading cause of anovulatory infertility in women (2, 3). In addition, women with PCOS have an increased risk of developing various chronic conditions, including cardiovascular disease, type 2 diabetes, and metabolic syndrome (2, 4, 5). For infertile PCOS patients seeking conception, controlled ovarian hyperstimulation (COH) represents a critical step in assisted reproductive technology (ART) (6). However, PCOS patients often exhibit heightened sensitivity to gonadotropin (Gn) stimulation, predisposing them to ovarian hyperstimulation syndrome (OHSS) (7). At the same time, their oocyte and embryo quality may be compromised, ultimately resulting in suboptimal live birth rates (8). Therefore, elucidating the underlying neuroendocrine regulatory mechanisms during COH in PCOS patients and identifying reliable biomarkers to predict ovarian responsiveness and pregnancy outcomes are of great clinical importance for achieving individualized COH management in PCOS.

Precise regulation of the hypothalamic–pituitary–ovarian axis (HPOA) is fundamental to normal follicular development and ovulation (9). The core pathophysiological alteration in PCOS involves dysfunction of the HPOA, primarily characterized by dysregulated GnRH release and excessive pulsatile secretion of luteinizing hormone (LH) (10). Kisspeptin neurons located in the hypothalamic arcuate nucleus serve as a key regulatory component of the GnRH pulse generator (11). Evidence from PCOS animal models has demonstrated that hyperactivation of Kisspeptin neurons directly drives abnormally frequent LH pulses and subsequent hyperandrogenemia (12), suggesting that Kisspeptin may play a central role in the pathogenesis of PCOS. Our team’s previous research has found elevated serum kisspeptin levels in PCOS patients, and kisspeptin has been shown to participate in the regulation of pituitary hormone secretion and energy metabolism (13). During COH, dynamic changes in Kisspeptin levels may reflect ovarian function and the status of follicular development, both of which influence COH and pregnancy outcomes. However, studies investigating the temporal patterns of Kisspeptin fluctuations during COH and their predictive value remain limited.

Therefore, the present study aimed to systematically observe and analyze the dynamic changes in Kisspeptin levels among PCOS patients during COH, and to further explore their associations with COH outcomes and pregnancy results. This approach not only provides deeper insight into the reproductive physiological mechanisms of PCOS but also offers potential predictive and therapeutic targets to optimize COH treatment strategies in this population.

## 2 Methods

### 2.1 Study population

A total of 100 infertile patients who underwent *in vitro* fertilization and embryo transfer (IVF-ET) at the Reproductive Center of Tianjin Medical University General Hospital between October 2023 and December 2024 were enrolled in this study, including 50 patients with PCOS (PCOS group) and 50 control patients who underwent IVF-ET during the same period (control group). All participants underwent COH using the gonadotropin-releasing hormone antagonist (GnRH-A) protocol. Fresh embryo transfer or frozen–thawed embryo transfer was performed as appropriate. The frozen–thawed embryo transfer protocols included natural, artificial, and stimulated cycles.

#### 2.1.1 Inclusion criteria for the PCOS group

According to the Rotterdam criteria, a diagnosis of PCOS was established when at least two of the following three conditions were met, after excluding other causes of hyperandrogenism and ovulatory dysfunction: 1) Oligo-ovulation or anovulation; 2) Clinical and/or biochemical hyperandrogenism; 3) Polycystic ovarian morphology.

#### 2.1.2 Inclusion criteria for the control group

1) Female patients undergoing IVF treatment during the same period due to tubal or male factors; 2) Normal baseline endocrine parameters; 3) Regular menstrual cycles; 4) Bilateral ovaries with normal morphology on ultrasound, and no uterine abnormalities (e.g., uterine fibroids ≤ 3 cm, no intrauterine adhesions).

#### 2.1.3 Exclusion criteria

Patients with adrenal disorders; those with a documented history of thyroid dysfunction, those currently receiving thyroid-related medications, or those with serum TSH levels outside the normal range (0.5–4.5 mIU/L); patients who had used any hormonal medications (including those who had undergone a COH cycle) within three months before enrollment; and individuals with other systemic diseases or complications were excluded.

### 2.2 COH Protocol and sample collection

On the 2nd–3rd day of menstruation, patients received daily Gn at a dose of 150–225 IU/day. GnRH-A (0.25 mg/day) was administered on the 5th–7th day after initiating Gn stimulation, or when the leading follicle reached a diameter of 14 mm, or when LH levels ≥10 IU/L. When two to three dominant follicles reached a diameter of 18 mm and the mean estradiol (E_2_) concentration per mature follicle was between 200–300 ng/L, ovulation was triggered with either hCG (5,000–10,000 IU) or recombinant hCG (rhCG, 250 μg). Oocyte retrieval was performed 36 hours later via transvaginal ultrasound-guided aspiration.

Peripheral venous blood samples (5 mL) were collected at four time points during the COH cycle: the day of starting Gn treatment (dGn), the fifth day of Gn stimulation (dGn5), the day of hCG injection (dhCG), the day of oocyte pickup (dOPU). follicular fluid (FF) was collected from follicles with a diameter ≥18 mm on dOPU. The expression levels of human Kisspeptin were measured using an ELISA kit (Cat. No. E-EL-H6099, Elabscience). All experimental results (n=3) were expressed as mean ± standard deviation (SD).

### 2.3 Statistical analysis

Statistical analyses were performed using SPSS version 27.0. Quantitative data with a normal distribution were expressed as mean ± SD, and comparisons between two groups were conducted using independent-samples t-tests, while comparisons among multiple groups were analyzed using one-way ANOVA. Quantitative data with a non-normal distribution were expressed as median (interquartile range) [M (IQR)], with group comparisons performed using the Mann–Whitney U test (for two groups) or the Kruskal–Wallis H test (for multiple groups). Categorical variables were presented as frequencies (percentages). Pearson’s correlation coefficient was used to assess the strength of linear relationships between continuous variables. Receiver operating characteristic (ROC) curve analysis was applied to evaluate the predictive performance of variables for binary outcomes. A two-tailed *P* value < 0.05 was considered statistically significant.

### 2.4 Ethics

All procedures performed were in accordance with the ethical standards of the institutional review board and with the 1964 Helsinki Declaration and its later amendments or comparable ethical standards. The study was approved by the Ethics Committee of Tianjin Medical University General Hospital (IRB2023-KY-332). Written informed consent was obtained from all patients for enrollment in this study.

## 3 Results

### 3.1 Comparison of baseline characteristics between the two groups

A total of 100 patients undergoing IVF-ET were included in this study, comprising 50 patients in the PCOS group and 50 in the control group. Comparison of baseline characteristics revealed that the PCOS group had significantly higher antral follicle count (AFC), basal anti-Müllerian hormone (AMH) levels, basal follicle-stimulating hormone (FSH) levels, and basal LH levels compared with the control group (*P* < 0.001, *P* < 0.001, *P* = 0.002, *P* = 0.014, *P* = 0.003, respectively). However, there were no significant differences between the two groups in terms of age, body mass index (BMI), previous pregnancies, basal E_2_ levels, basal progesterone (P) levels, or basal prolactin (PRL) levels (all *P*>0.05). (See **Table 1**).

**Table 1:** Comparison of general characteristics of patients.

Item	PCOS group	Control group	*P*
Age, years	31.78 ± 4.32	33.00 ± 3.24	0.113
BMI (kg/m^2^)	23.36 ± 3.75	23.07 ± 2.52	0.655
Previous pregnancies	0.38 ± 0.73	0.68 ± 1.08	0.106
AFC	24.90 ± 5.00	14.34 ± 5.22	<0.001
Basal AMH (ng/ml)	8743.56 ± 3260.85	2526.39 ± 1396.90	<0.001
Basal E_2_(pg/ml)	30.95 ± 12.73	32.05 ± 12.65	0.665
Basal P(ng/ml)	0.36 ± 0.35	0.29 ± 0.13	0.195
Basal T(ng/ml)	37.04 ± 15.18	28.60 ± 11.18	0.002
Basal FSH (mIU/ml)	5.99 ± 1.44	6.73 ± 1.49	0.014
Basal LH (mIU/ml)	5.05 ± 4.50	3.04 ± 1.12	0.003
Basal LH/Basal FSH	0.82 ± 0.63	0.47 ± 0.19	<0.001
Basal PRL (ng/ml)	17.26 ± 8.03	19.44 ± 8.84	0.198

BMI, Body Mass Index; AFC, Antral Follicle Count; AMH, Anti-mullerian Hormone; E_2_, Estradiol; P, Progesterone; T, Testosterone; FSH, follicle stimulating hormone; LH, Luteinizing Hormone; PRL, Prolactin.

### 3.2 Comparison of Kisspeptin levels at four time points between the two groups

At all four time points (dGn, dGn5, dhCG, and dOPU), serum and FF Kisspeptin levels in the PCOS group were significantly higher than those in the control group (all *P* < 0.001). Within-group comparisons of Kisspeptin levels at different time points revealed that, in the PCOS group, both serum and FF Kisspeptin levels at dOPU were significantly higher than those at dGn (*P* < 0.001). Similarly, in the control group, FF Kisspeptin levels at dOPU were higher than those at dGn, with a statistically significant difference (P<0.001). (See **Table 2**).

**Table 2:** Level of Kisspeptin in serum and FF during COH.

Item	PCOS group	Control group	*P*
dGn serum (pg/ml)	128.55 ± 61.69	72.26 ± 31.15	<0.001
dGn5 serum (pg/ml)	90.70 ± 47.24	58.73 ± 19.50	<0.001
dhCG serum (pg/ml)	142.53 ± 54.97	78.30 ± 37.31	<0.001
dOPU serum (pg/ml)	186.48 ± 65.44^***^	97.98 ± 46.49	<0.001
dOPU FF (pg/ml)	576.83 ± 175.85^***^	251.21 ± 110.91^***^	<0.001

Asterisks represent comparison of Kisspeptin levels at each time point vs. baseline (dGn). ***P < 0.001.

### 3.3 Correlation between Kisspeptin levels at different time points and baseline hormone levels

**Figure 1:**
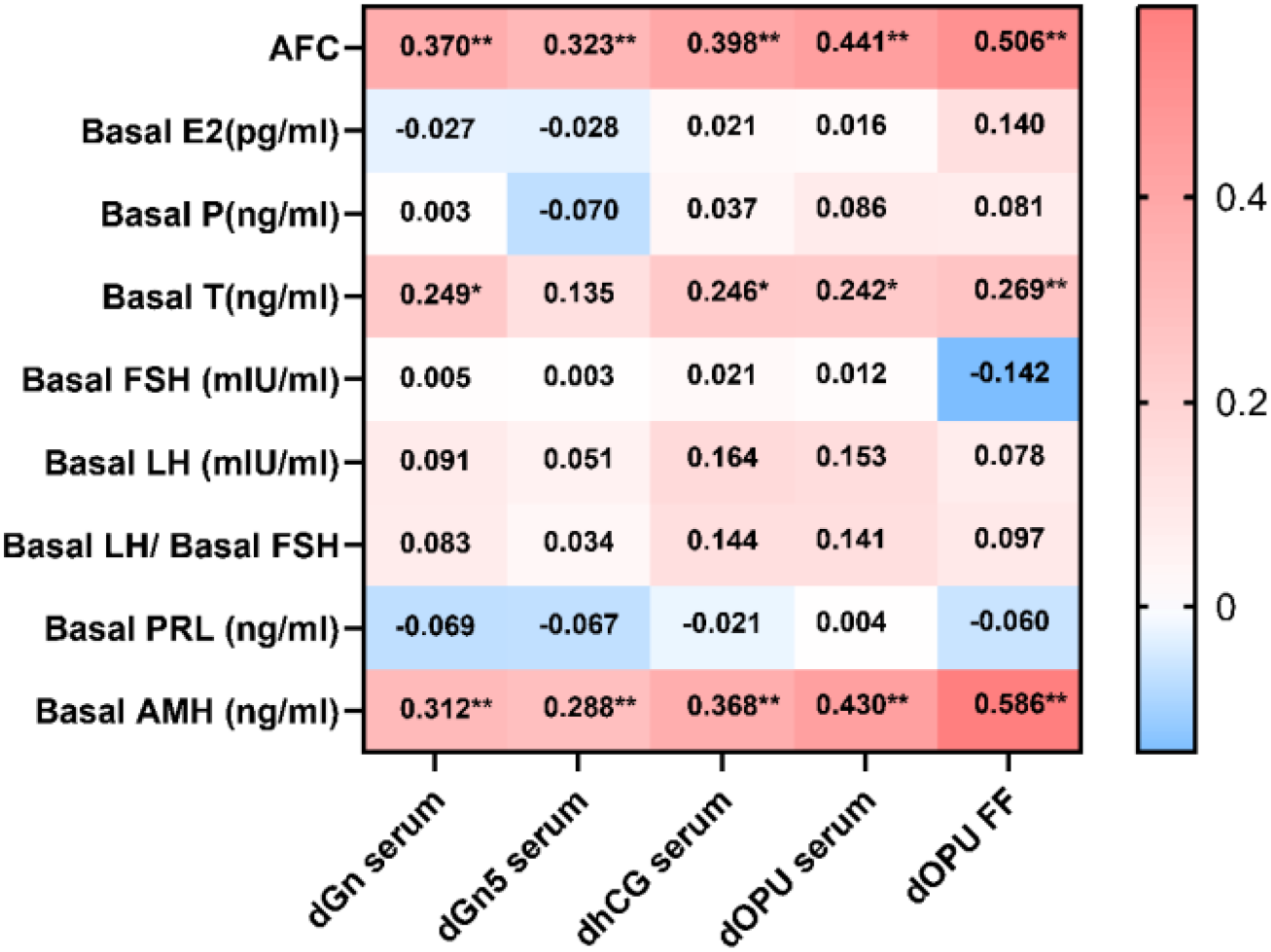
Correlation analysis between Kisspeptin levels and baseline hormonal parameters at different time points in all patients. The heatmap illustrates the Pearson correlation coefficients between serum and FF Kisspeptin levels at various time points and baseline hormonal parameters in all patients. The color gradient indicates the direction and strength of the correlations (see legend), with red representing positive correlations and blue representing negative correlations. *P* < 0.05; *P* < 0.01.

Pearson correlation analysis was performed to evaluate the associations between serum and FF Kisspeptin levels at different time points and baseline hormonal levels. Serum Kisspeptin levels at all four time points (dGn, dGn5, dhCG, and dOPU), as well as FF Kisspeptin levels at dOPU, showed positive correlations with AFC, basal T, and basal AMH levels. (Correlation coefficients are presented in **Figure 1**).

### 3.4 Comparison of COH outcomes between the two groups

The COH outcomes of the two groups were compared. The PCOS group showed significantly higher E_2_ levels on hCG day, number of retrieved oocytes, number of two pronuclei (2PN) embryos, and number of available embryos compared with the control group (all *P* < 0.001). However, the top-quality embryo (TQE) rate in the PCOS group was significantly lower than that in the control group (*P* < 0.001). No significant differences were observed between the two groups in total Gn dosage, duration of COH, 2PN rate, or the absolute number of TQE (all *P* > 0.05). (See **Table 3**).

**Table 3:** Comparison of COH outcomes of patients.

Item	PCOS group	Control group	*P*
Gonadotropins dosage (IU)	2360.50 ± 983.32	2417.88 ± 735.50	0.742
Duration of COH(d)	10.34 ± 2.14	9.84 ± 1.58	0.188
E_2_ on hCG day(pg/ml)	4216.5 ± 2341.08	2390.13 ± 1413.05	<0.001
Retrieved oocytes	24.96 ± 12.42	12.42 ± 5.41	<0.001
2PN of fertilization	14.80 ± 7.05	7.98 ± 3.68	<0.001
2PN rate (%)	62.21 ± 16.17	65.97 ± 16.76	0.275
Available embryos	14.22 ± 7.19	7.92 ± 3.69	<0.001
TQE	6.19 ± 4.84	4.53 ± 2.87	0.052
TQE rate (%)	39.30 ± 22.08	51.38 ± 21.54	<0.001

COH, Controlled Ovarian Hyperstimulation; 2PN, two pronuclei; TQE, Top-Quality Embryo.

### 3.5 Correlation between kisspeptin levels and COH outcomes

**Figure 2:**
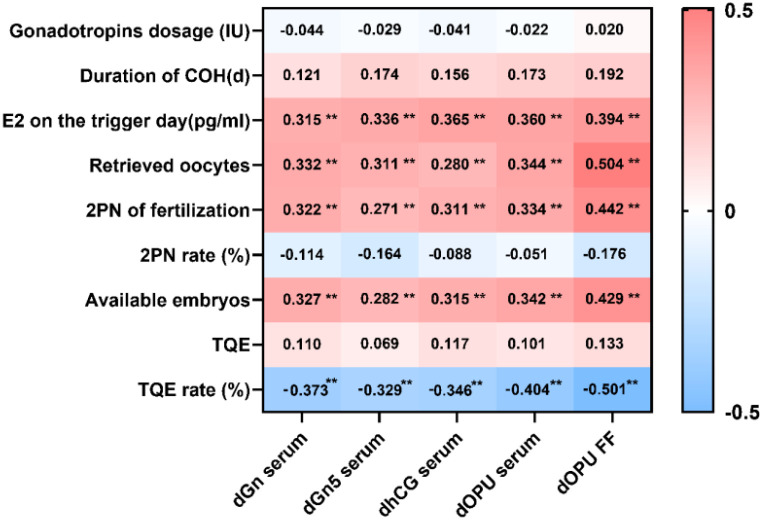
Correlation analysis between Kisspeptin levels and COH outcomes at different time points in all patients. The heatmap illustrates the Pearson correlation coefficients between serum and FF Kisspeptin levels at various time points and COH outcomes in all patients. The color gradient indicates the direction and strength of the correlations (see legend), with red representing positive correlations and blue representing negative correlations. *P* < 0.01.

Pearson correlation analysis was performed to evaluate the relationships between Kisspeptin levels in serum and FF and COH outcomes. Serum Kisspeptin levels at all four time points and FF Kisspeptin levels at dOPU were positively correlated with E_2_ levels on the trigger day, number of retrieved oocytes, number of 2PN embryos, and number of available embryos. Conversely, they were negatively correlated with the TQE rate. No statistically significant correlations were observed between Kisspeptin levels and total Gn dosage, duration of COH, 2PN rate, or TQE. (Correlation coefficients are presented in **Figure 2**).

### 3.6 Comparison of cumulative pregnancy rates between the two groups

This study further followed up on the pregnancy outcomes of both groups and compared their cumulative pregnancy rates. The cumulative pregnancy rate was 40.00% in the PCOS group and 60.00% in the control group. The cumulative pregnancy rate in the PCOS group was significantly lower than that in the control group (χ² = 4.00, *P* = 0.046). (See **Table 4**).

**Table 4:** Comparison of cumulative pregnancy rates between the two patient groups.

Groups	Samples	Outcomes		χ² test	
		Pregnancy	Not pregnancy	χ²	*P*
PCOS	50	40.00%(20/50)	60.00%(30/50)	4.00	0.046
Control	50	60.00%(30/50)	40.00%(20/50)		
Sum	100	50	50		

### 3.7 Comparison of embryo transfer profiles between the two groups

**Figure 3:**
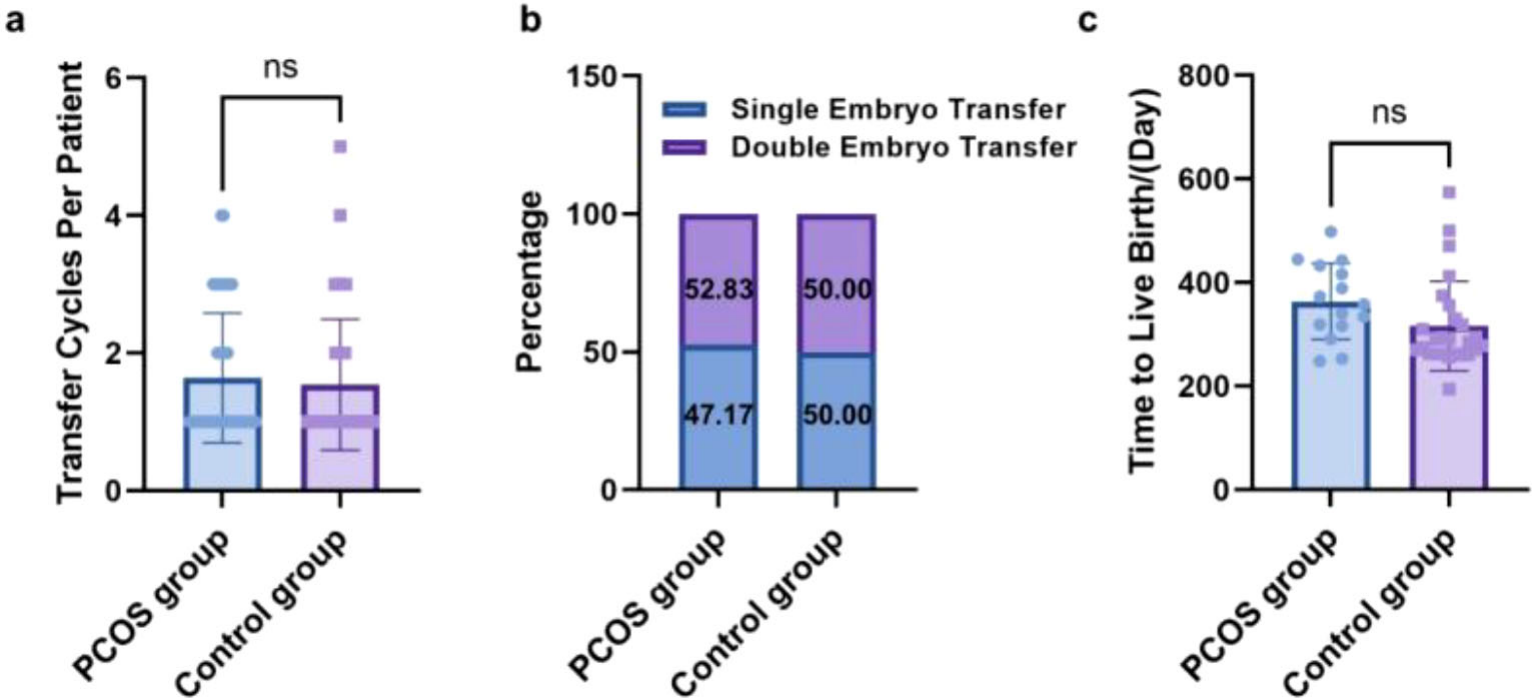
Bar graph showing the comparison of transfer profiles between two groups. **(a)** Number of transfer cycles per patient; **(b)** Proportions of single- and double-embryo transfers per cycle; **(c)** Time to live birth (TTLB).

As presented in **Figure 3**, in the PCOS group, 50 patients underwent 82 embryo transfer cycles (1.64 ± 0.94 per patient), while the control group underwent 77 cycles (1.54 ± 0.95 per patient), with no significant difference (*P* = 0.539) (**Figure 3a**). The proportions of single- and double-embryo transfers were also comparable (both *P* > 0.05) (**Figure 3b**), indicating that the observed difference in pregnancy outcomes is not confounded by transfer characteristics. The time to live birth (TTLB) was longer in the PCOS group (364 ± 74 days vs. 316 ± 86 days), but the difference did not reach statistical significance (*P* = 0.139) (**Figure 3c**).

### 3.8 Comparison of live birth rates between the two groups

Based on the pregnancy outcomes, this study further followed both groups through to live birth. As of October 2025, the PCOS group underwent 82 embryo transfer cycles, resulting in 14 live births, while the control group underwent 77 transfer cycles, resulting in 26 live births. Using the formula “Live birth rate = (number of live birth cycles/number of transfer cycles) × 100,” the live birth rate was calculated to be 17.07% in the PCOS group and 33.77% in the control group. The live birth rate in the PCOS group was significantly lower than that in the control group (χ² = 6.00, *P* = 0.014). (See **Table 5**).

**Table 5:** Comparison of live birth rates between the two patient groups.

Groups	Times	Outcomes		χ² test	
		Live birth	Not live birth	χ²	*P*
PCOS	82	17.07%(14/82)	82.93%(68/82)	6.00	0.014
Control	77	33.77%(26/77)	66.23%(51/77)		
Sum	159	40	119		

### 3.9 Correlation between kisspeptin and pregnancy outcomes

**Figure 4:**
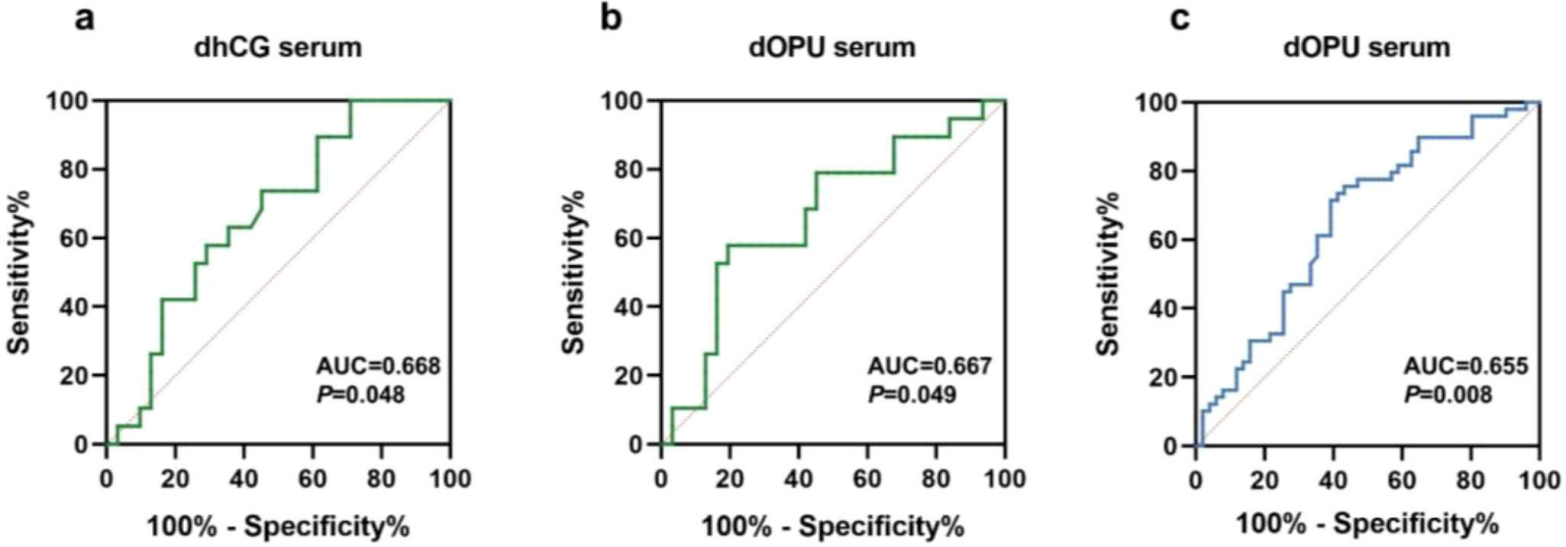
Kisspeptin levels predict pregnancy success. **(a)** Kisspeptin on the hCG day in the control group; **(b)** Kisspeptin on the OPU day in the control group; **(c)** Kisspeptin on the OPU day in all patients.

After completing the collection of pregnancy outcome data, we performed ROC analyses separately in the PCOS and control groups. No significant predictive value for pregnancy outcomes was observed for Kisspeptin levels at any time point within the PCOS group. In contrast, serum Kisspeptin measured on the dhCG (AUC = 0.668, *P* = 0.048; cut-off 81.96 pg/mL, sensitivity 0.579, specificity 0.710, **Figure 4a**) and on the dOPU (AUC = 0.667, *P* = 0.049; cut-off 125.0 pg/mL, sensitivity 0.579, specificity 0.807, **Figure 4b**) in the control group both showed predictive ability. Given the limited sample size (n=50 per group) and the associated risk of false-negative or false-positive findings, we extended the analysis to the whole cohort (n=100) to assess whether Kisspeptin could serve as a universal biomarker in the general assisted reproduction population. This whole-cohort ROC analysis revealed that both serum and follicular fluid Kisspeptin levels at various time points exhibited significant predictive performance for pregnancy outcomes. Among these, serum Kisspeptin on dOPU provided the best predictive value (AUC = 0.655, 95% CI: 0.547–0.763), with a cut-off value of 135.18 pg/mL, sensitivity of 0.39, and specificity of 0.71 (**Figure 4c**). Collectively, these findings suggest that serum Kisspeptin may serve as a potential biomarker for individualized prediction of pregnancy outcomes in COH patients.

## 4 Discussion

This study, designed as a prospective cohort study, aims to predict the outcomes of COH and pregnancy by dynamically monitoring changes in Kisspeptin levels during the COH process, thereby helping to alleviate reproductive difficulties in patients with PCOS.

In PCOS patients, dysfunction of the HPOA leads to follicular development arrest at the antral follicle stage, resulting in the accumulation of antral follicles (14). Previous studies have found that both AMH levels and AFC in PCOS patients are significantly higher than those in normal individuals, and that the amount of AMH secreted per follicle may also be greater (15). AMH is mainly secreted by antral follicles and reflects ovarian reserve capacity, and its level is considered to be positively correlated with AFC (16). The results of this study showed that AFC and AMH levels in the PCOS group were significantly higher than those in the control group, which is consistent with the pathophysiological characteristics of PCOS. In addition, baseline T and LH levels in the PCOS group were significantly higher than those in the control group, further supporting the typical features of hyperandrogenemia and an increased LH/FSH ratio in PCOS patients (12). However, in PCOS with insulin resistance (IR) rat model, endogenous Kisspeptin expression in ovarian granulosa cells is decreased, whereas our study shows elevated serum and follicular fluid Kisspeptin in normal-weight PCOS patients (17). We propose that this discrepancy reflects distinct regulatory states under different disease stages and metabolic burdens: the former represents local decompensation under severe IR, while the latter represents an early stage with enhanced central drive but preserved local reserve. This may be related to dysregulation of the HPOA in PCOS patients, leading to increased frequency and amplitude of LH secretion pulses. Persistently elevated LH levels subsequently cause excessive androgen secretion by ovarian theca cells (12). However, there were no significant differences between the two groups in age, BMI, previous pregnancies, baseline E_2_ level, baseline P level, or baseline PRL level, indicating that the PCOS group and the control group were comparable in these baseline characteristics, thus excluding the potential confounding effects of these factors on the study results.

Kisspeptin, a hypothalamic neuropeptide encoded by the KISS1 gene, not only regulates the HPOA within the central nervous system but also exerts direct local regulatory effects on follicular maturation in the ovary (18). Previous studies have found that serum Kisspeptin levels in PCOS patients are elevated compared with those in the control group (19), and the same phenomenon was observed in this study. This phenomenon may be associated with dysfunction of the HPOA in PCOS patients (18). In PCOS mouse models, the activity of hypothalamic Kisspeptin neurons is significantly enhanced, leading to abnormally increased LH pulse secretion; targeted inhibition of Kisspeptin neurons can reverse this effect (12). This mechanism may also exist in PCOS patients. Specifically, PCOS patients often exhibit abnormal pulsatile secretion of GnRH, resulting in elevated LH levels and an increased LH/FSH ratio. Since Kisspeptin is an upstream regulator of GnRH neurons and can directly stimulate GnRH release, the elevated Kisspeptin levels observed in PCOS patients may represent a compensatory response to HPOA dysfunction, aimed at modulating abnormal hormone levels by enhancing GnRH secretion.

A notable finding in this study was that Kisspeptin levels positively correlated with LH but not with FSH. This observation, while seemingly counterintuitive, is consistent with the physiological characteristics of Kisspeptin-mediated gonadotropin regulation. Clinical studies have demonstrated that Kisspeptin administration stimulates a robust increase in LH pulses, whereas FSH levels remain relatively stable (20). Similarly, in PCOS-like mouse models, hyperactivity of Kisspeptin neurons is directly associated with abnormally elevated LH pulse frequency, with no synchronous fluctuations in FSH (12). Mechanistically, Kisspeptin primarily regulates the frequency and amplitude of GnRH pulses; LH secretion is highly pulse-dependent, whereas FSH secretion is largely constitutive and subject to slower feedback regulation by inhibin, activin, and estradiol (21, 22). Therefore, the absence of a significant Kisspeptin-FSH correlation in our PCOS cohort likely reflects these fundamental differences in gonadotropin regulatory dynamics.

In addition, previous studies have shown that Kisspeptin exhibits distinct cyclic variations during the normal menstrual cycle: levels are low during the first five days of the cycle, show a first rise on day 11 (when the dominant follicle reaches a diameter of 1–2 cm), and a second smaller rise on day 14; such fluctuations have been observed in both serum and urine (23). A similar pattern of fluctuation was observed in this study. Kisspeptin levels in each group showed dynamic changes during the COH process, specifically presenting a statistically significant increase in Kisspeptin concentration on dOPU compared with dGn. This may be related to the regulation of GnRH pulse frequency by Kisspeptin neurons during dominant follicle development, leading to the formation of the LH surge (24).

Previous studies have shown that during COH, patients with PCOS exhibit a more intense response to Gns, characterized by a higher number of follicles at various developmental stages. Consequently, the number of retrieved oocytes in PCOS patients is usually greater than that in the control group; however, follicle size and maturity often display marked heterogeneity (24). This excessive and uneven follicular development is directly associated with OHSS (6). In addition, time to live birth in women with PCOS is often longer than that in non-PCOS women (25), which may be related to the prolonged duration of controlled ovarian stimulation (26) and the lower embryonic developmental potential in PCOS patients (27). In this study, no difference in time to live birth was observed between the PCOS and control groups, which may be attributed to the individualized use of the GnRH-A protocol among PCOS patients. Therefore, the COH process in PCOS populations should focus more on optimizing oocyte quality, reducing the risk of OHSS caused by follicular developmental heterogeneity, shortening IVF cycle duration, and achieving pregnancy as early as possible.

As an important regulatory factor of HPOA, Kisspeptin may exacerbate ovarian hyper-responsiveness to Gn, leading to asynchronous follicular development and oocyte maturation disorders (28), and may serve as an indicator for evaluating oocyte quality. However, previous studies have lacked correlation analyses between serum and FF Kisspeptin levels and COH outcomes. Through univariate correlation analysis, this study revealed the dynamic changes of Kisspeptin during the COH cycle and its association with clinical outcomes. The results showed that the number of retrieved oocytes, 2PN and TQE in the PCOS group were higher than those in the control group, whereas the TQE rate was lower, which can be explained by the high follicle count but asynchronous development and follicular maturation disorder in PCOS. Moreover, serum and FF Kisspeptin levels were correlated with key COH outcome indicators (such as the number of retrieved oocytes, available embryos, and TQE), suggesting that Kisspeptin may cooperatively regulate ovarian response through both the HPOA and local ovarian paracrine mechanisms (29). Kisspeptin-mediated activation of GnRH neurons to regulate follicular development has been demonstrated in mouse models (18). In this study, a prospective cohort design further verified the association between Kisspeptin levels and follicular development and maturation indicators during the COH cycle, particularly the predictive capacity of FF Kisspeptin levels for the TQE rate, which may provide a new target for optimizing ovulation induction protocols in PCOS patients. In summary, Kisspeptin exhibits a complex dynamic pattern during the COH cycle, and its correlation with COH outcomes not only reflects ovarian reserve function but may also reveal potential regulatory mechanisms underlying oocyte maturation.

Several studies have investigated the reproductive outcomes of patients with PCOS. A meta-analysis involving 104 studies reported significant differences in reproductive outcomes between women with PCOS and the normal population, showing increased risks of miscarriage, gestational diabetes, gestational hypertension, preeclampsia, and cesarean section (30). In this study, it was found that although the PCOS group had higher numbers of retrieved oocytes and 2PN, their pregnancy rate and live birth rate were both lower than those of the control group, suggesting that the developmental potential of oocytes in the PCOS group may be impaired. Furthermore, even after obtaining viable embryos through COH, the pregnancy rate in the PCOS group remained significantly lower than that in the control group, indicating that extra-ovarian factors may influence pregnancy outcomes. The extra-ovarian mechanisms affecting pregnancy outcomes in PCOS patients are complex and may involve multiple factors, including metabolic dysfunction (31), impaired endometrial receptivity (32), and chronic inflammation (33).

As a neuropeptide, Kisspeptin not only plays a fundamental role in regulating the female HPOA but also directly participates in key reproductive processes. It is widely expressed in the placenta and plays an important role in regulating endometrial receptivity (34) and embryo implantation (35), and may serve as a risk stratification biomarker for early pregnancy loss (36). A recent review by Zhang et al. further pointed out that excessively high Kisspeptin levels during pregnancy in PCOS patients ultimately increase the risk of pregnancy complications such as gestational diabetes mellitus and preeclampsia (37). Against this background, the present study focused on the dynamic changes of Kisspeptin during COH and its predictive value for pregnancy outcomes. We found that Kisspeptin levels in the PCOS group were significantly higher than those in the control group and were closely associated with decreased oocyte quality and adverse pregnancy outcomes. Moreover, ROC curve analysis showed that serum Kisspeptin levels measured at dGn, dGn5, dhCG, and dOPU could predict pregnancy outcomes in the COH population, and follicular fluid Kisspeptin maintained a similar predictive capacity. However, the dynamic changes and mechanisms of action of Kisspeptin during pregnancy still require further investigation. Therefore, future research should aim to longitudinally track Kisspeptin levels in IVF patients throughout the entire process from ovarian stimulation to pregnancy, in order to elucidate its independent and combined effects at different stages.

This study has certain innovative and practical value. Unlike previous cross-sectional studies, this study provides longitudinal characterization of kisspeptin dynamics during COH in PCOS patients, including concurrent serum and follicular fluid measurements, a predictive threshold for pregnancy outcomes, and its association with live birth. Although the predictive efficacy of kisspeptin alone is moderate, its integration into a multifactor model may have clinical value. Specifically, serum kisspeptin on dOPU with a cut-off of 135.18 pg/mL could serve as an auxiliary biomarker to identify PCOS patients at higher risk of adverse pregnancy outcomes, guiding elective freeze-all and subsequent frozen-thawed embryo transfer in clinical practice, and facilitating patient stratification for research on novel COH protocols or therapies targeting the hypothalamic-pituitary-ovarian axis.

This study has several limitations. First, the relatively small sample size may have limited the statistical power, potentially leading to false-negative results. Especially, due to the limited sample size (n=50 per group), subgroup ROC analyses are exploratory despite whole−cohort covariate−adjusted analyses. Moreover, PCOS is a highly heterogeneous disorder. A recent large-scale study using unsupervised cluster analysis identified four reproducible PCOS subtypes, including a hyperandrogen subtype, an obesity subtype, a high-sex hormone-binding globulin subtype, and a high-luteinizing hormone-anti-Müllerian hormone subtype (38). The BMI of the PCOS cohort in our study (23.36 ± 3.75 kg/m²) falls within the normal range, which may reflect the enrollment of a specific subtype and possible selection bias. Therefore, the conclusions of this study are primarily applicable to normal-weight PCOS patients, and caution should be exercised when generalizing these findings to overweight or obese PCOS populations. In addition, the ELISA kit used in this study measured only total Kisspeptin levels and could not distinguish between different isoforms (such as Kisspeptin-10 and Kisspeptin-54), which may possess distinct biological activities. The present study cannot fully establish a direct causal relationship between Kisspeptin and live birth rate, and its predictive efficacy (AUC = 0.655) is modest, suggesting that Kisspeptin alone is insufficient to guide clinical decision-making. Future large-scale, multicenter prospective studies incorporating additional biomarkers are needed to validate its clinical value.

In summary, the dynamic changes in Kisspeptin levels may have potential significance for predicting COH outcomes in PCOS populations; however, its clinical utility as an independent predictive indicator remains to be validated through studies with larger sample sizes, longer follow-up periods, and more refined detection methods.

## Data Availability

The raw data supporting the conclusions of this article will be made available by the authors, without undue reservation.
